# GSSA-YOLOM-Based Foreign Object and Conveyor Belt Deviation Detection

**DOI:** 10.3390/s26041381

**Published:** 2026-02-22

**Authors:** Zuguo Chen, Jiayu Liu, Yimin Zhou, Yi Huang, Chenghao Liang

**Affiliations:** 1 Sanya Institute of Hunan University of Science and Technology, Sanya 572024, China; 2 School of Information and Electrical Engineering, Hunan University of Science and Technology, Xiangtan 411201, China; 3 Hunan Provincial Key Laboratory of Intelligent Control and Maintenance for Complex Systems, Hunan University of Science and Technology, Xiangtan 411201, China; 4 Shenzhen Institutes of Advanced Technology, Chinese Academy of Sciences, Shenzhen 518055, China

**Keywords:** multi-task learning, conveyor belt, foreign object detection, belt deviation detection

## Abstract

The safety of belt conveyor operation is of great importance during coal conveyance. This paper proposes a multi-task-based GSSA-YOLOM algorithm for monitoring the state of belt conveyors, which utilizes segmentation head to detect foreign objects and belt deviation, thereby balancing the trade-offs among multiple tasks. The detection neck is responsible for multi-scale feature fusion by incorporating the Asymptotic Feature Pyramid Network (AFPN) to achieve enhanced spatial perception. Then, Groupwise Separable Convolution (GSConv) is further introduced to simplify the network architecture, reducing computational complexity while maintaining sufficient detection accuracy for edge device deployment. Moreover, the SlideLoss and Soft-NMS functions are integrated to reduce the rate of false positives and missed detections. Comparison experiments were conducted, and the results indicate that the proposed GSSA-YOLOM model can improve mAP@50 by 3.4% compared with the baseline model while reducing the number of parameters by 27%, thereby satisfying coal mine safety monitoring requirements.

## 1. Introduction

The underground coal mine transportation plays an important role in coal production. As an essential tool in modern coal mining operations, belt conveyors have gained widespread popularity due to their high efficiency and reliability in continuous material transport [1,2]. However, their operational safety remains susceptible to key failures such as belt deviation, foreign object carriage, and roller shedding [3]. Such faults not only lead to production interruptions and economic losses but also may trigger catastrophic safety incidents, particularly in underground environments. Timely identification and correction of abnormal phenomena are crucial for extending conveyor service life, ensuring production stability, and minimizing accident risks [4].

Traditional manual inspection methods suffer from low efficiency, poor environmental adaptability, and vulnerability to visual fatigue. Although a range of advanced sensors and monitoring systems have been deployed to improve inspection performance [5], they often fail to provide immediate alerts. Existing machine vision-based detection methods still face technical challenges in complex underground conditions, including uneven illumination, notable target scale variations, and insufficient real-time performance. Therefore, it is necessary to explore efficient and lightweight abnormal detection methods for underground coal mine belt conveyors to enhance intelligent safety capabilities and promote unmanned mine construction.

The advancement in deep learning has substantially improved the robustness and accuracy of conveyor belt monitoring systems. Among various anomalies, structural defects in conveyor belts (such as misalignment and roller detachment) and the transportation of foreign objects present two major challenges. Correspondingly, visual perception algorithms concentrate on two primary tasks: perception of the conveyor belt state and identification of foreign objects carried on the conveyor. For instance, DeepLabv3+ has been successfully employed for pixel-level segmentation of different conveyor components [6], achieving high segmentation accuracy and precise boundary extraction. Advanced attention mechanisms have been incorporated into segmentation networks to further improve overall performance [7]. Based on the segmented belt edges, the relative distance between them can be computed, which serves as a critical indicator for detecting misalignment issues such as belt deviation [8]. The object identification methods have two main categories: two-stage and single-stage detection frameworks. The two-stage detectors, exemplified by Faster R-CNN [9], have been effectively utilized for non-coal foreign object recognition owing to their high detection accuracy. Lv et al. [10] further expanded this approach by integrating the VGG16 network, to enhance the recognition accuracy of the challenging categories, i.e., gangue and iron rods.

In contrast, single-stage detection algorithms like the YOLO series [11,12,13] and SSD [14] are widely preferred due to their faster inference speeds, high precision, and lower computational costs, such as malicious URL detection using a backbone and bidirectional LSTM [15]. YOLOv5 and YOLOv7 have been implemented for foreign object detection on conveyor belts, enabling real-time tracking of non-coal materials with notable effectiveness [16,17].

However, most existing methods treat belt segmentation and object detection independently. And relying on a single feature is prone to noise and environmental disturbances [18]. This results in multi-task detection algorithms like DR-YOLOM proposed by Li et al. [19], which can simultaneously perform strip-like edge segmentation, coal flow segmentation and foreign object identification, but suffer from large model parameters and high hardware requirements, making them difficult to be deployed on the edge computing platforms.

**Figure 1:**
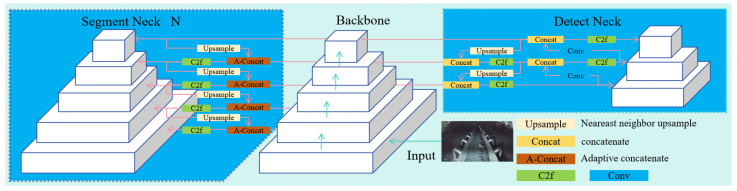
The architecture of the A-YOLOM network [20].

**Figure 2:**
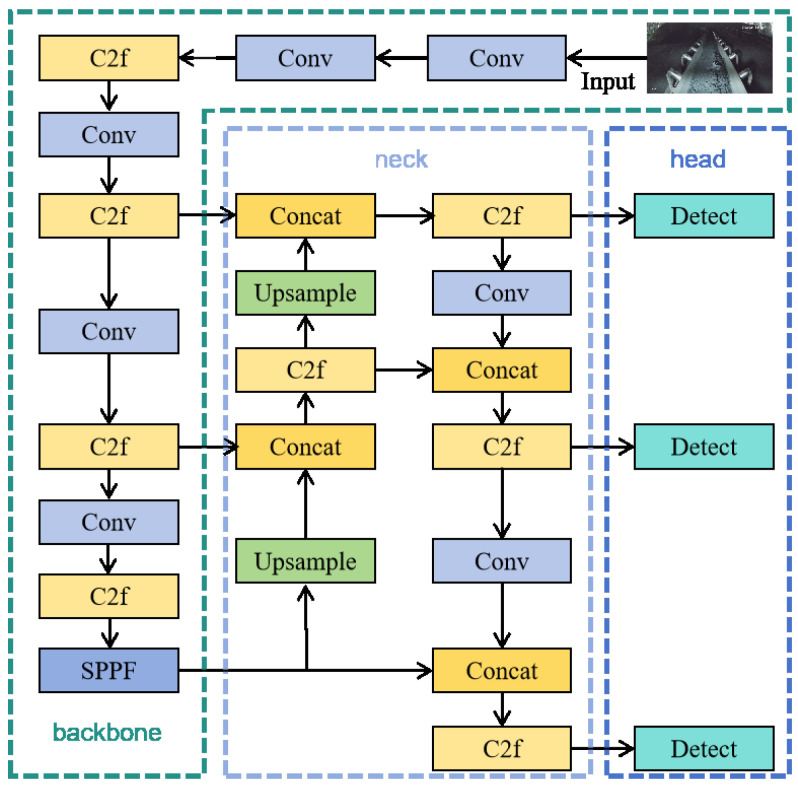
The diagram of the YOLOv8 network.

To address these challenges, this paper proposes a multi-task learning network named GSSA-YOLOM with the A-YOLOM baseline model [20]. Here, GSSA represents the integration of GSConv, SlideLoss, Soft-NMS and AFPN. As illustrated in Figure 1, the backbone of A-YOLOM originates from the YOLOv8 architecture, possessing high accuracy and fast processing speed [21]. The used frame in YOLOv8 is illustrated in Figure 2 where the backbone network can enhance the feature extraction and representation capabilities. C2f refers to the cross-stage partial module used in the YOLOv8 backbone. The feature fusion neck comprises the Feature Pyramid Network (FPN) and Path Aggregation Network (PAN) to effectively integrate multi-scale features [22]. Through upsampling and downsampling operations, YOLOv8 can facilitate the fusion of high-level semantic information and low-level spatial details [23], thereby improving the detection performance across various scaled objects.

However, while YOLOv8 excels in either detection or segmentation tasks individually, the multi-task learning framework A-YOLOM (n) can simultaneously perform both detection and segmentation tasks. Its nano variant (n) is selected here due to the shallowest depth and minimal computational burden among the existing models [24]. The neck module structure consists of two independent pathways with corresponding task-specific heads to accommodate the dual tasks of segmentation and object detection. The model remains the original YOLOv8 neck and object detection head for the object detection, while an adaptive feature concatenation module is introduced between the backbone and neck for segmentation. This module can handle multiple feature resolutions and dynamically determine whether to propagate or fuse features. The segmentation head comprises multiple convolutional layers for context aggregation, followed by a deconvolution layer to restore the original image resolution and generate pixel-level binary masks.

The contribution of the manuscript is summarized as follows:

(1) A high-precision multi-task object detection model, GSSA-YOLOM, is proposed to achieve simultaneous abnormal object detection and biased monitoring in coal convey transportation.

(2) The Groupwise Separable Convolution is applied to optimize the backbone and detection neck convolutional modules so as to reduce the model size and computational cost, while the asymptotic FPN is introduced to mitigate the information loss and degradation during multi-stage feature transmission processes.

(3) The SlideLoss function is further applied to balance the disparity between direct and challenging samples, and the non-maximum suppression function is also employed to enhance the model detection accuracy.

(4) A series of comparison experiments have been performed to verify the efficacy of the proposed GSSA-YOLOM model in coal mine safety monitoring system with higher robustness.

The remainder of this manuscript is organized as follows: Section 2 introduces the design of the conveyor belt inspection system and the corresponding improvement measures. Section 3 details the proposed fault detection methods and decision criteria for belt misalignment. Section 4 evaluates the system performance through experimental results and analysis conducted under various operating conditions. Section 5 concludes the paper and discusses potential future work.

## 2. The Proposed GSSA-YOLOM Method

**Figure 3:**
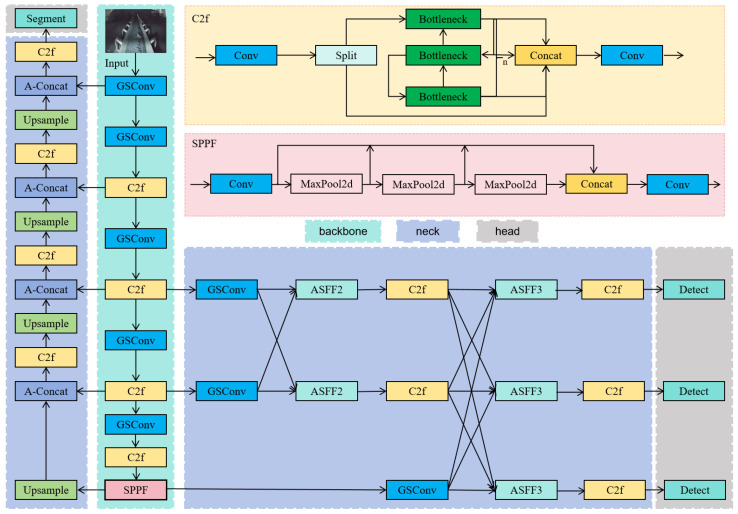
The proposed GSSA-YOLOM network.

The GSSA-YOLOM (seen in Figure 3) multi-task detection algorithm is proposed to detect the objects, i.e., roller, metal rods, foreign objects and belt edge for real-time belt conveyor monitoring, where a shared backbone network performs the feature extraction for both the segmentation and object detection tasks.

First, the Asymptotic FPN (AFPN) [25] module is incorporated into the detection neck to improve the extraction of crucial details from the coal mine images. Groupwise Separable Convolution (GSConv) optimization [26] is applied to both the backbone and detection neck convolutional modules to achieve a lightweight structure without performance degradation. To enhance focus on hard samples (e.g., foreign objects), SlideLoss [27] is incorporated into the binary cross-entropy loss; meanwhile, Non-maximum suppression (Soft-NMS) [28] is applied to suppress false detections due to occlusion and improve final accuracy.

### 2.1. AFPN Module

In coal transportation scenarios with the conveyor belts, objects are in diversified sizes, thus the AFPN is incorporated to enhance the multi-scale feature fusion capability.

**Figure 4:**
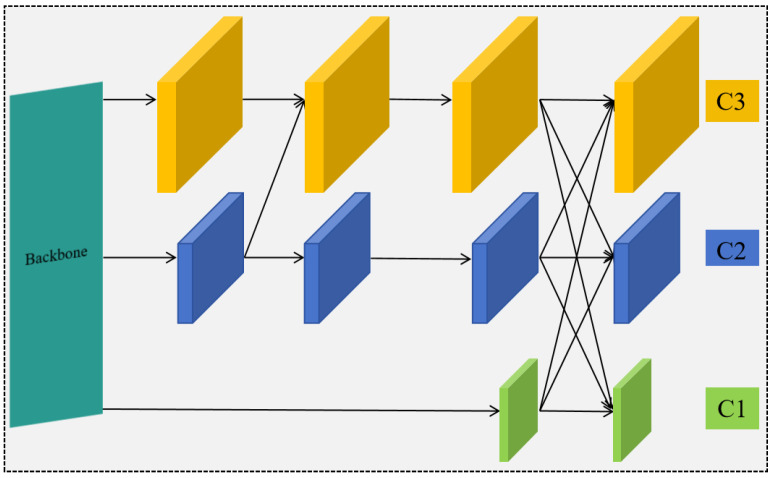
The AFPN structure.

Figure 4 displays the architecture of the AFPN with a three-layer progressive feature fusion network with C2f and ASFF modules. After the feature extraction via the backbone network, three features with different scales are obtained, ranging from small to large scope as 1C, 2C and 3C. The 2C and 3C features are first fused, followed by the fusion of the more abstract small-scale feature 1C. Features of different scales are aligned in dimension through upsampling and downsampling, while the features of the same scale are directly mapped through lateral connections. This progressive fusion of the adjacent hierarchical features can effectively mitigate the semantic gaps between non-adjacent levels while reducing the feature information loss during the fusion process. The enhanced integration capability of the network can increase the precision and recall in identifying small targets.

### 2.2. GSConv Module

**Figure 5:**
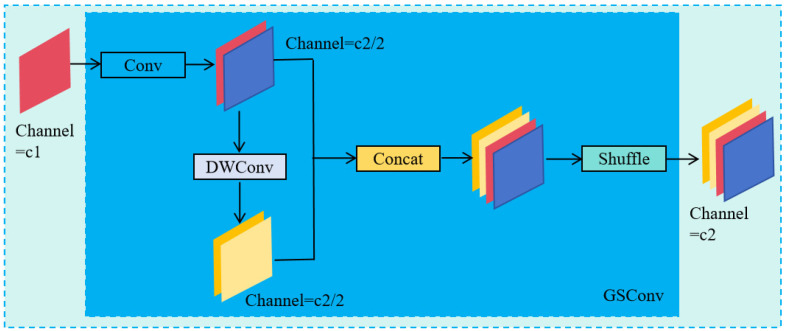
The GSConv structure.

Figure 5 illustrates the structure of the GSConv module to extract the features with reduced computational cost, storage requirements and parameters amount. Initially, the GSConv applies a standard convolution to the input, producing a feature map with half of the C2 channels, thereby enhancing its feature representation. Next, this intermediate map is processed via a depthwise separable convolution to create another map with the same number of channels so as to lower the computational complexity. These two maps are then concatenated to form a combined feature map with C2 channels. Finally, the channel shuffling is used to evenly distribute the feature information, resulting in the final output feature map with the C2 channels requisite.

### 2.3. SlideLoss Module

Due to the large quantity disparity between the rollers and foreign objects, the SlideLoss function is applied to refine the standard cross-entropy loss. It can adaptively learn two key parameters, a positive-sample (μ) and a negative-sample threshold, which can guide the model to emphasize the difficult-to-classify samples. μ is defined as the mean Intersection over Union (IoU) across all positive samples and the sliding weighting function, which assigns emphasis to samples near the decision boundary. By assigning higher loss weights in the neighborhood of μ, the penalty of misclassifying ambiguous examples can be written as,

(1) $$f\left(x\right)=\left\{\begin{array}{cc} 1, & x\leq \mu -0.1\\ e^{1-\mu }, & \mu -0.1\leq x\leq \mu \\ e^{1-x}, & x\geq \mu \end{array}\right.$$

where *x* denotes the IoU value between a predicted bounding box and its corresponding ground-truth box.

### 2.4. Soft-NMS Module

Soft-NMS is used to eliminate the redundant bounding boxes which overlap with the highest-confidence box (*M*), exceeding a set threshold ($N_{t}$). However, it can also lead to missed detections in densely populated scenes. Here, Soft-NMS is introduced to adjust the confidence scores of the overlapped low-confidence boxes via a Gaussian decay function, written as,

(2) $$s_{i}=\left\{\begin{array}{cc} s_{i}, & \mathrm{IOU}\left(M,b_{i}\right)<N_{t}\\ s_{i}\left(1-\mathrm{IOU}\left(M,b_{i}\right)\right), & \mathrm{IOU}\left(M,b_{i}\right)\geq N_{t} \end{array}\right.$$

where *M* is the candidate box with the highest current prediction score and $b_{i}$ is the candidate box to be processed.

## 3. The GSSA-YOLOM-Based Fault Detection

The proposed coal conveyor belt inspection system employs a distributed architecture comprising a host unit responsible for visual processing and an embedded controller managing sensor data. The host unit is built around an NVIDIA Jetson Nano module, which executes the real-time GSSA-YOLOM algorithm for image analysis. In parallel, an STM32F407 microcontroller (STMicroelectronics, Geneva, Switzerland) functions as the embedded controller, handling data acquisition from auxiliary sensors. A dual-mode industrial camera is mounted on the inspection robot and oriented toward the conveyor belt surface. As the robot travels along the belt, the camera continuously captures video streams at a maximum resolution of 2688×1520 pixels, ensuring reliable operation under both day-light and low-light conditions. The processed detection results and system status are transmitted via a wireless network to a cloud-based monitoring platform.

### 3.1. Fault Detection in Conveyor Belts

**Figure 6:**
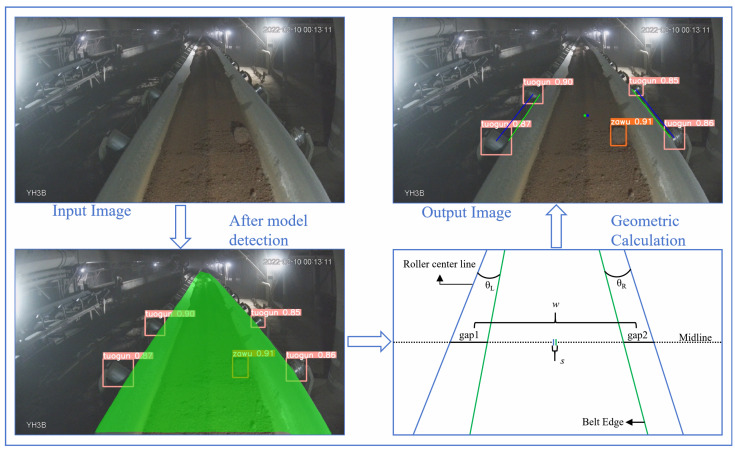
Illustration of the object detection in coal conveyance scene.

The core diagnostic system is the geometric reasoning pipeline built upon the outputs of the GSSA-YOLOM model. As illustrated in Figure 6, the model can provide instance segmentation masks for the conveyor belt and bounding box detections for rollers (labeled as “tuogun”) and foreign objects (labeled as “zawu”). These perceptual outputs form the basis for quantitatively assessing two critical fault types, i.e., belt misalignment and foreign object intrusion, where the abnormal objects are directly identified by the GSSA-YOLOM detector. The system constructs a polygonal zone from the detected boundaries of the four idlers, and an immediate alarm is triggered when the center of gravity of any foreign object lies within this polygon, indicating the presence of an abnormality in the critical zone between the idler and the conveyor belt.

### 3.2. Conveyor Belt Deviation Assessment

Belt misalignment is assessed by analyzing the geometric relationship between the segmented belt edges and the detected roller positions in an inspection robot scenario. The camera is rigidly mounted on the robot platform and maintains a stable pose relative to the conveyor belt during routine patrol, which provides a consistent geometric reference for deviation estimation. The overall procedure consists of three main stages, as illustrated in Figure 6.

(1) Preprocessing and feature extraction.The region of interest (ROI) is first defined by cropping 10% from each side of the conveyor belt segmentation mask to eliminate potential interference from image borders. Within this ROI, the Canny edge detection algorithm is applied to extract precise pixel-level belt edge points.

(2) Geometric feature calculation. Based on the detected left and right roller pairs, their centroids are first computed. The straight line connecting these two centroids is defined as the reference roller centerline, which is shown as the blue line in Figure 6. Subsequently, the extracted belt edge points are divided into left and right sets, and linear least-squares fitting is applied to fit the belt edge lines on both sides, which are shown as the green lines in Figure 6. In addition, the geometric centroid of the belt segmentation mask within the range of the blue line (the green point in Figure 6) and the geometric centroid of the roller pairs (the blue point in Figure 6) are calculated.

(3) Deviation quantification and fault criteria. The severity of belt misalignment is quantified using two complementary geometric metrics:

- Angular deviation. Two deviation angles, denoted as $\theta_{L}$ and $\theta_{R}$, are defined as the angles between the left and right fitted belt edge lines and the reference roller centerline, respectively. If either $\theta_{L}$ or $\theta_{R}$ exceeds the predefined threshold $\theta_{\mathrm{th}}$, the belt is identified as locally skewed, indicating a twisting fault.
- Lateral displacement. The lateral displacement distance *s* is defined as the perpendicular distance between the belt centroid and the reference roller centerline. Here, *w* denotes the nominal gap between the belt edge and the roller under normal operating conditions. A scaling factor α (0<α<1) is introduced to define an allowable displacement threshold. When the condition s>αw is satisfied, the belt is considered to experience global lateral misalignment.

Through the above three-stage procedure, both local belt skewing and global lateral misalignment can be effectively quantified, providing a reliable basis for subsequent fault diagnosis and experimental validation.

## 4. Experimental and Result Analysis

### 4.1. Experimental Setting

The experiment dataset is the Conveyor Belt Foreign Object Detection and Material Deviation Detection Challenge, which is publicly released by iFLYTEK for fair performance evaluation, containing 585 images from various real-world operational scenarios. Four primary categories of the objects are included, i.e., metal rods, rollers, belt edges and general debris, commonly encountered foreign objects like plastic bottles and paper. Table 1 lists the involved experiment parameters.

The Stochastic Gradient Descent (SGD) optimizer is employed during training to reduce the risk of convergence to suboptimal local minima. The training parameters are set as follows: the initial learning rate is 0.01, the momentum coefficient is 0.937, and the batch size is 16. Mosaic data augmentation is applied throughout the training process and the model is trained for 300 epochs. Table 2 summarizes the training configuration.

Since the proposed model is designed for multi-task learning, including object detection and semantic segmentation, the dataset requires multi-format annotations. The public dataset provides bounding box annotations for foreign objects and metal rods. Bounding box annotations for rollers are manually added using the Labelme software, while polygon-based masks are annotated to delineate the edges of the conveyor belt for the segmentation task.

The dataset is randomly partitioned into training, validation and test sets with a ratio of 7:2:1 while maintaining a similar distribution of object categories across different subsets. This split strategy ensures fair training, hyperparameter tuning and unbiased performance evaluation.

In this paper, the detection performance for the rollers and metal rods on the conveyor belt is evaluated with precision, recall and mean average precision (mAP). For the segmentation of belt edges, mIoU (mean Intersection over Union) is adopted as the primary metric. To further assess the model efficiency, the number of parameters and Giga Floating-Point Operations Per Second (GFLOPS) are calculated for each model, written as,

(3) $$\begin{array}{cc} \mathrm{Precision} & =\frac{TP}{TP+FP} \end{array}$$

(4) $$\begin{array}{cc} \mathrm{Recall} & =\frac{TP}{TP+FN} \end{array}$$

(5) $$\begin{array}{cc} {\mathrm{AP}}_{i} & =\int_{0}^{1}P_{i}\left(R\right)\;dR \end{array}$$

(6) $$\begin{array}{cc} \mathrm{mAP} & =\frac{1}{N}\sum_{i=1}^{N}{\mathrm{AP}}_{i} \end{array}$$

(7) $$\begin{array}{cc} \mathrm{mIoU} & =\frac{1}{C}\sum_{c=1}^{C}{\mathrm{IoU}}_{c} \end{array}$$

where ${\tilde{P}}_{i}\left(R\right)$ denotes the interpolated precision–recall curve for class *i*, *N* is the number of detection classes and *C* is the number of segmentation classes.

### 4.2. Ablation Experiment

To evaluate the detection performance of the proposed network architecture, the A-YOLOM algorithm is employed as the baseline model and further modified with the incorporation of several key modifications: (A) replacing the detection head with AFPN, (B) adopting SlideLoss as the loss function, (C) substituting traditional NMS with Soft-NMS, and (D) replacing standard convolutional layers with GSConv modules.

Table 3 summarizes the results of the ablation experiments performed on the validation dataset, with all the models trained under the same configuration outlined in Section 4.1. The ablation results reveals that the architectural improvements can emphasize the lightweight design while maintaining or improving the detection accuracy.

Groups 2 and 3, incorporating SlideLoss and Soft-NMS individually, can improve the mAP@50 by 0.4% and 0.7%, respectively, with the same parameters amount. Group 4, which combines both SlideLoss and Soft-NMS, can achieve a 2.1% mAP@50 gain over the baseline model.

Further, the integration of AFPN module in Group 5 can reduce parameter amount but improve the mAP@50 by 0.4% compared to that of Group 4. Group 6, with all four enhancements, can achieve 89.9% mAP@50, maintain mIoU in segmentation and reduce 27% parameter amount.

**Figure 7:**
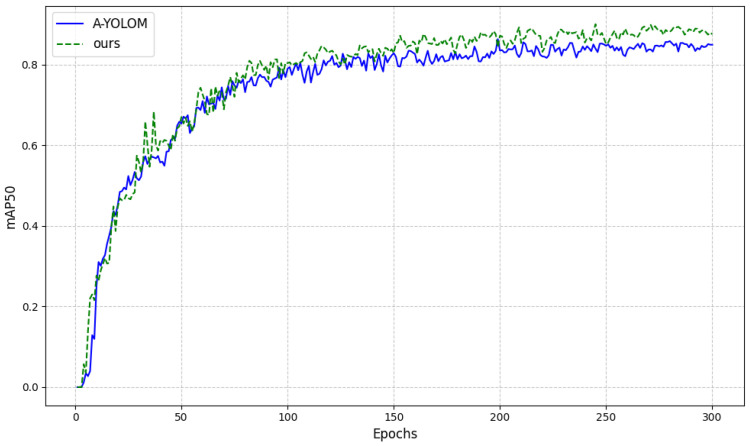
Comparison of mAP50 before and after the improvements.

Table 4 further reports the per-class detection AP and belt-edge segmentation mIoU under representative ablation settings. The results indicate that the proposed improvements primarily enhance the detection performance of foreign objects, which exhibit greater diversity and pose more challenges, while the belt-edge segmentation mIoU remains consistently high across all configurations, demonstrating the robustness of the proposed segmentation branch. The improvement in average detection accuracy is visually displayed in Figure 7.

Compared to the baseline A-YOLOM model, our proposed model demonstrates marked enhancements in mAP, parameter efficiency and computational performance. It is particularly well suited for deployment on edge devices with constrained computation power and lightweight framework.

### 4.3. Comparison Experiment

To evaluate the application capability of the proposed GSSA-YOLOM framework, the segmentation performance is compared with U-Net [29] and DeepLabV3+ [30]. For the object detection, the proposed method is evaluated against several models from the YOLO series. The comparation results are listed in Table 5.

Table 5 lists the detection performance, segmentation performance and model parameter efficiency between the proposed algorithm and existing mainstream models on the coal dataset. For the simultaneous tasks of segmentation and detection addressed in this study, the YOLO network requires deploying two distinct models on the embedded devices with doubled parameters compared to that of the proposed method. The comparison clearly demonstrates that the proposed algorithm can ensure simultaneous execution of segmentation and detection tasks.

**Figure 8:**
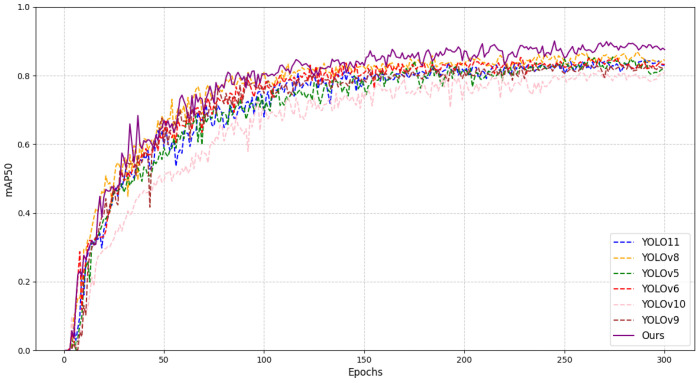
Detection mAP50 comparison.

**Figure 9:**
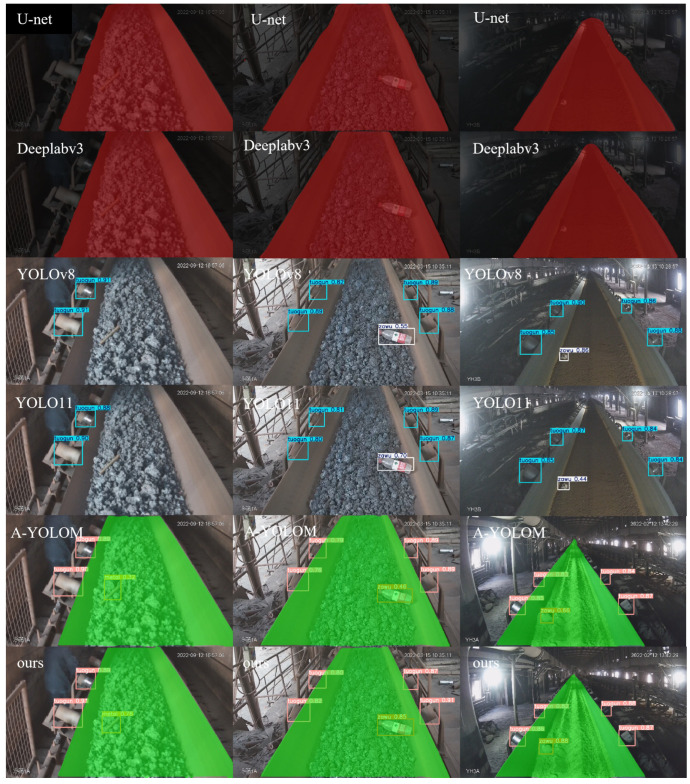
Partial detection results.

Figure 8 demonstrates that the proposed model achieves superior performance on the mAP50 metric compared to existing models. Figure 9 compares the qualitative detection and segmentation results of U-Net, DeepLabV3+, YOLOv8, YOLO11, A-YOLOM and the proposed method on the IFlytek public dataset. For clarity, only the four bottom rollers are shown. Three target categories are considered and the conveyor belt region is indicated using colored segmentation masks. U-Net and DeepLabV3+ produce blurred or incomplete belt boundaries under illumination variations and background interference. YOLOv8 and YOLO11 can localize rollers and foreign objects but often suffer from missed detections and inaccurate bounding boxes in cluttered scenes. Although A-YOLOM improves robustness, false positives and localization errors still occur. In contrast, the proposed method provides accurate roller localization and clear belt edge segmentation for reliable belt deviation assessment.

### 4.4. Belt Deviation Assessment

**Figure 10:**
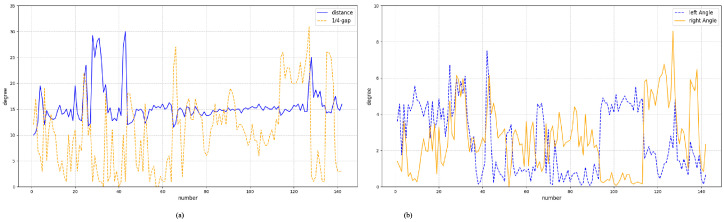
Experimental validation of belt deviation assessment. (**a**) Angular deviations $\theta_{L}$ and $\theta_{R}$ of the belt edges. (**b**) Lateral displacement with the threshold.

To quantitatively evaluate the proposed belt deviation assessment method, 177 test images with identical resolution were used for validation. A total of 30 images of severe deviation cases were excluded to focus on typical operating conditions and only four cases failed to detect belt deviation correctly. The lateral misalignment threshold was defined as one fourth of the reference half width with α=0.25. The measurement statistics are shown in Figure 10.

Figure 10a shows the angular deviations $\theta_{L}$ and $\theta_{R}$ of the belt edges, while Figure 10b shows the lateral displacement *s* with the threshold αw. The results demonstrate that the proposed method can reliably characterize belt deviation.

## 5. Discussion

This paper proposes a multi-task detection method, GSSA-YOLOM, for foreign object recognition and belt segmentation in belt conveyor systems. The algorithm incorporates several enhancements, including Soft-NMS, SlideLoss, AFPN and GSConv modules, which can collectively reduce false negatives and false positives in foreign object detection. It supports the simultaneous execution of segmentation and detection within a unified model to improve overall accuracy but with reduced amount parameters. The experimental results demonstrate that the proposed model can achieve higher mAP50 and segmentation task performance compared to other models.

Future work will focus on improving model accuracy by incorporating a broader range of fault categories, such as belt tears and idling, and by evaluating the generalization performance across a wider variety of conveyor systems and operating scenarios.

## Figures and Tables

**Table 1:** System configuration.

Component	Specification
Operating System	Ubuntu 20.04
Programming Language	Python 3.8
Deep Learning Frameworks	PyTorch 1.12.0
CUDA Version	11.3
GPU	NVIDIA GeForce RTX 3090

**Table 2:** Training configuration.

Component	Specification
Initial learning rate	0.01
Momentum coefficient	0.937
Batch size	16
Number of training epochs	300

**Table 3:** Results of the ablation experiments.

Model	A	B	C	D	P	R	mAP@50	Params	GFLOPS
					(%)	(%)	(%)	(M)	
Group 1	-	-	-	-	43.1	91.8	86.5	3.33	7.43
Group 2	-	✓	-	-	42.2	90.2	86.9	3.33	7.43
Group 3	-	-	✓	-	88.7	81.4	87.2	3.33	7.43
Group 4	-	✓	✓	-	88.5	83.2	88.6	3.33	7.43
Group 5	✓	✓	✓	-	89.7	84.0	89.0	2.637	6.92
Group 6	✓	✓	✓	✓	90.8	85.5	89.9	2.429	6.68

Note: ✓ indicates the inclusion of the corresponding module. (A) AFPN modules, (B) SlideLoss function, (C) Soft-NMS modules, and (D) GSConv modules.

**Table 4:** Per-class detection and segmentation performance under different ablation settings.

Model	Roller AP (%)	Foreign Object AP (%)	Metal Rod AP (%)	Belt Edge mIoU (%)
Group 1	99.4	68.8	91.3	98.9
Group 2	99.5	72.9	88.3	98.9
Group 3	99.4	71.6	90.7	98.9
Group 4	99.4	78	88.3	98.9
Group 5	99.5	75.6	91.9	98.9
Group 6	99.5	78.4	91.9	98.9

**Table 5:** Performance comparison of different models.

Model	Params (M)	mAP50 (%)	mIOU (%)
U-net	24.89	-	97.77
DeepLabV3+	5.81	-	98.48
YOLO11n	2.582	84.2	-
YOLOv10n	2.695	79.8	-
YOLOv8n	3.0	85	-
YOLOv5n	2.509	84.3	-
YOLOv6n	4.23	83.3	-
YOLOv9t	1.971	84.2	-
ours	2.429	89.9	98.9

## Data Availability

No new data were created or analyzed in this study.

## Abbreviations

AFPN	Adaptive Feature Pyramid Network
C2f	Conveyor-to-frame distance
CNN	Convolutional Neural Network
GFLOPS	Giga Floating-Point Operations Per Second
GSConv	Groupwise Separable Convolution
IoU	Intersection over Union
mAP	mean Average Precision
mIoU	mean Intersection over Union
NMS	Non-Maximum Suppression
ROI	Region of Interest
Soft-NMS	Soft Non-Maximum Suppression
SPPF	Spatial Pyramid Pooling-Fast
YOLO	You Only Look Once
